# Supplementary material for: Effectiveness of a reactive oral cholera vaccination during a cholera outbreak at the Douala New-Bell Central Prison in Cameroon
Source: PLoS Negl Trop Dis. 2025 Dec 29;19(12):e0013870. doi: 10.1371/journal.pntd.0013870 (PMC12774372; doi:10.1371/journal.pntd.0013870)
Supplement: S1 File — (PDF) [file pntd.0013870.s001.pdf]

	id	age	sex	penal	cell	diarrhoea	vomit	clinical	case	confirm
1	1	20	1	1	1	1	1	1	1	0
2	2	43	1	1	1	1	1	1	1	0
3	3	38	1	1	1	1	1	1	1	0
4	4	23	1	1	5	1	1	1	1	0
5	5	19	1	1	1	1	1	1	1	0
6	6	31	1	0	1	1	1	1	1	0
7	7	31	1	0	1	1	1	0	1	0
8	8	37	1	0	5	1	1	1	1	0
9	9	23	1	1	1	1	1	1	1	1
10	10	19	1	1	1	1	1	0	1	0
11	11	26	1	1	1	0	0	0	0	0
12	12	43	1	1	1	1	1	1	1	0
13	13	23	1	0	5	0	0	0	0	0
14	14	20	1	1	5	1	1	1	1	0
15	15	21	1	1	1	1	1	0	1	0
16	16	30	1	1	5	1	1	1	1	0
17	17	23	1	0	1	1	1	1	1	0
18	18	22	1	0	1	1	1	1	1	0
19	19	23	1	0	1	0	0	0	0	0
20	20	20	1	1	1	1	1	1	1	1
21	21	29	1	1	5	1	1	1	1	1
22	22	21	1	1	5	1	1	1	1	0
23	23	22	1	1	1	0	0	0	0	0
24	24	43	1	1	4	1	1	1	1	0
25	25	22	1	0	5	1	1	0	1	0
26	26	38	1	1	5	1	1	0	1	0
27	27	27	1	1	1	1	1	0	1	0
28	28	20	1	1	1	1	1	1	1	0
29	29	31	1	1	2	1	1	0	1	0
30	30	27	1	0	1	1	1	0	1	0
31	31	36	1	1	1	1	1	1	1	0
32	32	38	1	1	1	1	1	1	1	0
33	33	29	1	1	4	0	0	0	0	0
34	34	44	1	1	1	1	1	1	1	1
35	35	21	1	1	5	1	1	1	1	0
36	36	21	1	1	1	1	1	1	1	0
37	37	28	1	0	1	1	1	0	1	0
38	38	22	1	1	5	1	1	0	1	0
39	39	38	1	1	1	1	1	1	1	0
40	40	30	1	1	1	1	1	0	1	0
41	41	38	1	1	1	1	1	0	1	0
42	42	25	1	1	5	1	1	1	1	1
43	43	35	1	0	5	1	1	1	1	0
44	44	70	1	1	1	1	1	1	1	0
45	45	32	1	1	1	0	0	0	0	0
46	46	45	1	1	5	1	1	1	1	0
47	47	45	1	1	5	1	1	1	1	0
48	48	27	1	0	1	1	1	0	1	0
49	49	32	1	1	1	1	1	1	1	0
50	50	27	1	1	1	1	1	1	1	0

	id	age	sex	penal	cell	diarrhoea	vomit	clinical	case	confirm
51	51	32	1	1	1	1	1	1	1	0
52	52	33	1	1	1	1	1	1	1	0
53	53	21	1	1	1	1	1	1	1	0
54	54	29	1	1	5	1	1	1	1	0
55	55	33	1	1	1	1	1	1	1	0
56	56	32	1	1	1	1	1	1	1	0
57	57	26	1	1	1	1	1	1	1	0
58	58	29	1	1	1	1	1	1	1	0
59	59	35	1	1	1	1	1	1	1	0
60	60	19	1	1	5	1	1	0	1	0
61	61	34	1	0	5	1	1	0	1	0
62	62	26	1	1	1	1	1	1	1	0
63	63	23	1	1	5	1	1	0	1	0
64	64	21	1	1	1	1	1	0	1	0
65	65	19	1	1	1	1	1	0	1	0
66	66	28	1	1	1	1	1	0	1	0
67	67	20	1	0	1	1	1	0	1	0
68	68	20	1	0	1	1	1	0	1	0
69	69	25	1	0	1	1	1	1	1	0
70	70	30	1	0	1	1	1	1	1	0
71	71	34	1	0	1	1	1	1	1	0
72	72	19	0	0	4	1	1	0	1	0
73	73	19	1	1	1	1	1	0	1	0
74	74	31	1	1	5	1	1	0	1	0
75	75	30	1	1	4	1	1	0	1	0
76	76	33	1	1	5	1	1	0	1	0
77	77	32	1	1	1	1	1	1	1	0
78	78	33	1	1	5	1	1	0	1	0
79	79	26	1	1	1	1	1	0	1	0
80	80	23	0	1	3	1	1	0	1	0
81	81	35	1	1	1	1	1	0	1	0
82	82	39	0	1	3	1	1	0	1	0
83	83	25	1	1	5	1	1	0	1	0
84	84	31	1	1	1	1	1	0	1	0
85	85	42	1	1	1	1	1	0	1	0
86	86	34	1	1	1	1	1	0	1	0
87	87	24	1	1	1	0	0	0	0	0
88	88	31	1	1	5	1	1	0	1	0
89	89	32	1	1	5	1	1	1	1	0
90	90	22	1	1	5	1	1	1	1	0
91	91	34	1	0	1	1	1	1	1	0
92	92	30	1	1	5	1	1	1	1	0
93	93	31	1	1	5	1	1	0	1	0
94	94	35	1	1	5	0	0	0	0	0
95	95	27	1	0	5	1	1	0	1	0
96	96	24	1	1	1	0	0	0	0	0
97	97	35	1	1	1	1	1	0	1	0
98	98	20	1	1	1	1	1	0	1	0
99	99	26	1	1	5	1	1	0	1	0
100	100	35	1	1	1	0	0	0	0	0

	id	age	sex	penal	cell	diarrhoea	vomit	clinical	case	confirm
101	101	37	1	0	5	1	1	1	1	0
102	102	22	1	1	5	1	1	1	1	0
103	103	28	1	1	1	0	0	0	0	0
104	104	27	1	1	1	0	0	0	0	0
105	105	22	1	1	5	1	1	1	1	0
106	106	26	1	1	5	0	0	0	0	0
107	107	23	1	1	1	0	0	0	0	0
108	108	29	1	1	5	1	1	0	1	0
109	109	22	1	1	1	0	0	0	0	0
110	110	32	1	0	4	1	1	0	1	0
111	111	44	1	1	5	1	1	0	1	0
112	112	19	1	1	1	1	1	1	1	0
113	113	40	1	1	1	1	1	0	1	0
114	114	41	1	1	1	1	1	1	1	0
115	115	20	1	1	5	1	1	0	1	0
116	116	23	1	1	1	1	1	0	1	0
117	117	25	1	1	1	1	1	0	1	0
118	118	42	1	1	1	1	1	0	1	0
119	119	27	1	0	1	0	0	0	0	0
120	120	43	1	1	5	0	0	0	0	0
121	121	29	1	1	5	1	1	1	1	0
122	122	33	1	0	1	1	1	1	1	0
123	123	26	1	1	1	0	0	0	0	0
124	124	19	1	1	5	1	1	1	1	0
125	125	24	1	0	5	1	1	1	1	0
126	126	27	1	1	5	1	1	1	1	0
127	127	23	1	1	5	1	1	1	1	0
128	128	26	1	0	5	1	1	1	1	0
129	129	23	0	1	2	1	1	0	1	0
130	130	28	1	0	2	1	1	0	1	0
131	131	27	1	1	1	1	1	0	1	0
132	132	42	1	1	2	1	1	1	1	0
133	133	22	1	1	3	1	1	1	1	0
134	134	31	1	1	2	1	1	1	1	0
135	135	27	1	0	3	1	1	1	1	0
136	136	21	1	1	2	1	1	1	1	0
137	137	25	1	1	2	1	1	1	1	0
138	138	25	1	1	1	1	1	1	1	0
139	139	26	1	1	1	1	1	0	1	0
140	140	25	1	1	3	1	1	1	1	0
141	141	25	1	1	3	1	1	1	1	0
142	142	22	1	1	3	1	1	0	1	0
143	143	24	1	1	1	1	1	0	1	0
144	144	24	1	0	5	0	0	0	0	0
145	145	22	1	0	2	0	0	0	0	0
146	146	38	1	1	5	1	1	1	1	0
147	147	19	1	1	5	1	1	1	1	0
148	148	40	1	0	4	1	1	1	1	0
149	149	23	1	1	5	1	1	1	1	0
150	150	20	1	1	2	1	1	1	1	0

	id	age	sex	penal	cell	diarrhoea	vomit	clinical	case	confirm
151	151	35	1	1	3	1	1	1	1	0
152	152	38	1	1	1	1	1	1	1	0
153	153	27	1	1	3	1	1	1	1	0
154	154	30	1	1	3	1	1	1	1	0
155	155	33	1	1	5	1	1	1	1	0
156	156	29	1	1	1	0	0	0	0	0
157	157	33	1	1	4	1	1	0	1	0
158	158	30	1	1	5	1	1	1	1	0
159	159	27	1	0	5	1	1	1	1	0
160	160	23	1	1	5	1	1	0	1	0
161	161	24	1	0	5	1	1	1	1	0
162	162	23	0	1	2	0	0	0	0	0
163	163	27	1	1	5	1	1	0	1	0
164	164	30	0	1	4	1	1	0	1	0
165	165	30	1	1	5	1	1	1	1	0
166	166	24	1	1	4	1	1	1	1	0
167	167	24	1	0	3	1	1	1	1	0
168	168	25	1	1	5	1	1	0	1	0
169	169	29	1	1	5	1	1	1	1	0
170	170	28	1	1	5	1	1	1	1	0
171	171	21	1	1	5	1	1	1	1	0
172	172	31	1	1	5	1	1	0	1	0
173	173	37	1	1	5	1	1	0	1	0
174	174	24	1	1	1	1	1	1	1	0
175	175	42	1	1	5	1	1	1	1	0
176	176	25	1	1	5	1	1	1	1	0
177	177	25	1	0	5	1	1	0	1	0
178	178	25	1	1	5	1	1	1	1	0
179	179	38	1	1	3	1	1	0	1	0
180	180	30	1	1	1	1	1	0	1	0
181	181	24	1	1	1	1	1	0	1	0
182	182	65	1	1	3	1	1	0	1	0
183	183	28	1	0	2	1	1	0	1	0
184	184	32	1	0	1	1	1	0	1	0
185	185	21	1	0	5	1	1	0	1	0
186	186	49	1	1	5	1	1	0	1	0
187	187	23	1	1	1	1	1	0	1	0
188	188	27	1	1	1	1	1	1	1	0
189	189	36	1	1	5	1	1	1	1	0
190	190	25	1	0	5	1	1	1	1	0
191	191	36	0	1	2	1	1	1	1	0
192	192	25	1	0	5	1	1	1	1	0
193	193	28	1	1	1	1	1	1	1	0
194	194	28	1	0	5	1	1	0	1	0
195	195	23	1	1	1	1	1	0	1	0
196	196	26	0	1	2	1	1	1	1	0
197	197	34	0	1	3	1	1	0	1	0
198	198	34	0	1	2	1	1	0	1	0
199	199	37	1	1	5	1	1	0	1	0
200	200	23	1	0	1	1	1	0	1	0

	id	age	sex	penal	cell	diarrhoea	vomit	clinical	case	confirm
201	201	45	0	0	2	1	1	0	1	0
202	202	21	1	0	1	1	1	0	1	0
203	203	28	0	0	2	0	0	0	0	0
204	204	35	1	0	5	0	0	0	0	0
205	205	20	1	1	5	0	0	0	0	0
206	206	31	1	1	5	0	0	0	0	0
207	207	31	0	1	3	0	0	0	0	0
208	208	41	0	1	3	0	0	0	0	0
209	209	41	0	0	3	0	0	0	0	0
210	210	17	1	1	5	0	0	0	0	0
211	211	33	0	0	3	1	1	1	1	0
212	212	33	0	1	3	0	0	0	0	0
213	213	37	1	1	1	0	0	0	0	0
214	214	23	1	1	5	0	0	0	0	0
215	215	32	1	1	2	1	1	1	1	0
216	216	18	1	1	1	1	1	1	1	0
217	217	38	1	0	5	1	1	1	1	0
218	218	22	1	1	5	1	1	1	1	0
219	219	23	1	1	5	1	1	1	1	0
220	220	63	1	0	5	1	1	1	1	0
221	221	23	1	0	1	1	1	1	1	0
222	222	34	1	0	5	1	1	1	1	0
223	223	34	1	0	1	1	1	0	1	0
224	224	28	1	1	1	1	1	0	1	0
225	225	27	1	0	1	1	1	0	1	0
226	226	28	0	0	5	1	1	0	1	0
227	227	35	1	1	1	1	1	0	1	0
228	228	33	1	1	5	1	1	0	1	0
229	229	78	0	1	2	1	1	0	1	0
230	230	29	0	1	3	1	1	0	1	0
231	231	17	1	1	5	1	1	0	1	0
232	232	17	1	1	5	1	1	0	1	0
233	233	21	1	1	5	1	1	0	1	0
234	234	25	1	1	5	0	0	0	0	0
235	235	35	1	0	1	1	1	0	1	0
236	236	16	1	1	5	0	0	0	0	0
237	237	31	1	0	5	0	0	0	0	0
238	238	24	1	1	1	0	0	0	0	0
239	239	15	1	1	5	0	0	0	0	0
240	240	23	0	1	1	0	0	0	0	0
241	241	42	1	1	1	0	0	0	0	0
242	242	38	1	1	1	0	0	0	0	0
243	243	26	0	1	4	0	0	0	0	0
244	245	26	0	1	4	0	0	0	0	0
245	246	23	1	1	5	0	0	0	0	0
246	247	23	1	1	1	0	0	0	0	0
247	248	52	1	1	5	1	1	1	1	0
248	249	43	1	1	1	1	1	1	1	0
249	250	37	1	1	1	1	1	1	1	0
250	251	49	1	1	5	1	1	1	1	0

	id	age	sex	penal	cell	diarrhoea	vomit	clinical	case	confirm
251	252	45	1	1	1	1	1	1	1	0
252	253	63	0	1	2	1	1	1	1	0
253	254	13	1	1	5	1	1	0	1	0
254	255	30	0	1	2	1	1	0	1	0
255	256	45	1	1	1	1	1	0	1	0
256	257	39	1	0	1	1	1	0	1	0
257	258	33	1	1	5	1	1	0	1	0
258	259	32	1	0	5	1	1	1	1	0
259	260	36	1	1	1	1	1	0	1	0
260	261	24	1	1	5	1	1	0	1	0
261	262	26	1	0	1	1	1	0	1	0
262	263	25	1	1	5	1	1	0	1	0
263	264	26	1	1	1	1	1	0	1	0
264	265	31	1	1	1	1	1	1	1	0
265	266	46	1	1	5	1	1	0	1	0
266	267	43	1	1	4	1	1	0	1	0
267	268	24	1	1	5	1	1	0	1	0
268	269	22	1	1	5	1	1	1	1	0
269	270	38	1	1	2	1	1	1	1	0
270	271	23	1	1	3	1	1	1	1	0
271	272	33	1	1	4	1	1	0	1	0
272	273	30	0	1	2	1	1	1	1	0
273	274	23	0	1	3	1	1	1	1	0
274	275	28	0	1	2	1	1	1	1	0
275	276	30	0	0	2	1	1	1	1	0
276	277	61	0	1	3	1	1	1	1	0
277	278	28	1	1	1	1	1	1	1	0
278	279	24	1	1	5	1	1	1	1	0
279	280	25	0	1	2	1	1	0	1	0
280	281	18	0	1	2	1	1	0	1	0
281	282	21	0	1	2	1	1	0	1	0
282	283	29	0	1	2	1	1	0	1	0
283	284	31	0	1	2	1	1	0	1	0
284	285	31	1	1	1	1	1	0	1	0
285	286	30	0	1	3	1	1	0	1	0
286	287	24	0	1	2	1	1	0	1	0
287	288	38	0	1	2	1	1	0	1	0
288	289	24	1	1	1	1	1	0	1	0
289	290	25	1	1	1	1	1	0	1	0
290	291	26	1	1	1	1	1	0	1	0
291	292	33	0	1	2	1	1	0	1	0
292	293	29	1	1	5	1	1	0	1	0
293	294	22	1	1	2	1	1	0	1	0
294	295	32	0	1	3	1	1	0	1	0
295	296	40	0	1	2	1	1	1	1	0
296	297	27	0	1	2	1	1	1	1	0
297	298	24	0	1	3	1	1	1	1	0
298	299	26	1	0	5	1	1	1	1	0
299	300	25	1	1	5	1	1	1	1	0
300	301	27	1	1	5	1	1	1	1	0

	id	age	sex	penal	cell	diarrhoea	vomit	clinical	case	confirm
301	302	27	0	1	2	1	1	1	1	0
302	303	26	0	1	3	1	1	1	1	0
303	304	24	0	0	3	1	1	1	1	0
304	305	22	0	1	2	1	1	1	1	0
305	306	23	0	1	2	1	1	1	1	0
306	307	23	0	1	3	1	1	1	1	0
307	308	33	0	1	2	1	1	1	1	0
308	309	29	1	0	5	1	1	1	1	0
309	310	35	0	0	1	1	1	1	1	0
310	311	29	1	1	1	1	1	1	1	0
311	312	28	0	1	2	1	1	1	1	0
312	313	43	0	1	2	1	1	1	1	0
313	314	25	0	0	2	1	1	1	1	0
314	315	22	1	1	5	1	1	1	1	0
315	316	22	1	1	5	1	1	1	1	0
316	317	37	0	1	2	1	1	1	1	0
317	318	48	1	1	5	1	1	1	1	0
318	319	21	1	1	1	1	1	1	1	0
319	320	22	1	1	1	1	1	1	1	0
320	321	40	1	1	5	1	1	1	1	0
321	322	27	0	1	2	1	1	1	1	0
322	323	42	0	1	3	1	1	1	1	0
323	324	33	0	1	3	1	1	1	1	0

	ocv	doses	ors	meo	hosp	time	referal	dead	loperamide	cel
1	0	0	1	1	1	1	1	0	1	2
2	0	0	1	0	1	2	1	0	1	2
3	0	0	1	0	1	2	1	0	1	2
4	0	0	1	0	1	2	1	0	0	2
5	0	0	1	0	1	2	1	0	0	2
6	0	0	1	0	1	2	1	0	0	2
7	0	0	1	0	1	1	1	0	1	2
8	0	0	1	1	1	1	1	0	0	2
9	0	0	1	1	1	1	1	1	1	2
10	0	0	1	1	0	.	0	0	0	2
11	1	2	0	0	0	.	0	0	0	2
12	0	0	1	0	1	2	1	0	0	2
13	1	1	0	0	0	.	0	0	0	2
14	0	0	1	0	1	2	1	0	1	2
15	0	0	1	0	1	1	1	0	1	2
16	0	0	1	0	1	2	1	0	0	2
17	0	0	1	0	1	2	1	0	1	2
18	0	0	1	0	1	2	1	0	1	2
19	1	1	0	0	0	.	0	0	0	2
20	0	0	1	1	1	1	1	1	0	2
21	0	0	1	1	1	1	1	1	1	2
22	0	0	1	1	1	1	1	0	1	2
23	1	2	0	0	0	.	0	0	0	2
24	0	0	1	0	1	2	1	0	0	2
25	0	0	1	1	0	.	0	0	0	2
26	0	0	1	0	1	1	1	0	0	2
27	0	0	1	1	0	.	0	0	0	2
28	0	0	1	0	1	2	1	0	1	2
29	0	0	1	0	1	1	1	0	0	1
30	0	0	1	1	0	.	0	0	0	2
31	0	0	1	0	1	2	1	0	1	2
32	0	0	1	0	1	2	1	0	1	2
33	1	2	0	0	0	.	0	0	0	2
34	0	0	1	0	1	2	1	1	0	2
35	0	0	1	0	1	2	1	0	0	2
36	0	0	1	1	1	1	1	0	0	2
37	0	0	1	1	0	.	0	0	0	2
38	0	0	1	1	0	.	0	0	1	2
39	0	0	1	0	1	2	1	0	0	2
40	0	0	1	1	0	.	0	0	0	2
41	0	0	1	1	0	.	0	0	0	2
42	0	0	1	0	1	2	1	1	0	2
43	0	0	1	0	1	2	1	0	1	2
44	0	0	1	1	1	1	1	0	1	2
45	1	1	0	0	0	.	0	0	0	2
46	0	0	1	0	1	2	1	0	1	2
47	0	0	1	0	1	2	1	0	0	2
48	0	0	1	1	0	.	0	0	0	2
49	0	0	1	0	1	2	1	0	1	2
50	0	0	1	0	1	2	1	0	0	2

	ocv	doses	ors	meo	hosp	time	referal	dead	loperamide	cel
51	0	0	1	0	1	2	1	0	1	2
52	0	0	1	0	1	2	1	0	1	2
53	0	0	1	0	1	2	1	0	1	2
54	0	0	1	0	1	2	1	0	1	2
55	0	0	1	0	1	2	1	0	0	2
56	0	0	1	0	1	2	1	0	1	2
57	0	0	1	0	1	2	1	0	1	2
58	0	0	1	0	1	2	1	0	0	2
59	0	0	1	0	1	2	1	0	0	2
60	0	0	1	1	0	.	0	0	0	2
61	0	0	1	0	1	1	1	0	1	2
62	0	0	1	0	1	2	1	0	0	2
63	0	0	1	1	0	.	0	0	1	2
64	0	0	1	1	0	.	0	0	1	2
65	0	0	1	1	0	.	0	0	1	2
66	0	0	1	1	0	.	0	0	1	2
67	0	0	1	1	0	.	0	0	1	2
68	0	0	1	1	0	.	0	0	0	2
69	0	0	1	0	1	2	1	0	0	2
70	0	0	1	0	1	2	1	0	1	2
71	0	0	1	0	1	2	1	0	1	2
72	0	0	1	1	0	.	0	0	1	2
73	0	0	1	1	0	.	0	0	1	2
74	0	0	1	1	0	.	0	0	1	2
75	0	0	1	1	0	.	0	0	1	2
76	0	0	1	1	0	.	0	0	1	2
77	0	0	1	0	1	2	1	0	1	2
78	0	0	1	1	0	.	0	0	0	2
79	0	0	1	1	0	.	0	0	0	2
80	1	1	1	1	0	.	0	0	1	1
81	0	0	1	1	0	.	0	0	1	2
82	0	0	1	1	0	.	0	0	1	1
83	0	0	1	1	0	.	0	0	1	2
84	0	0	1	1	0	.	0	0	0	2
85	0	0	1	1	0	.	0	0	1	2
86	0	0	1	1	0	.	0	0	1	2
87	1	2	0	0	0	.	0	0	0	2
88	0	0	1	1	0	.	0	0	0	2
89	0	0	1	1	1	1	1	0	1	2
90	0	0	1	1	1	1	1	0	0	2
91	0	0	1	1	1	1	1	0	1	2
92	0	0	1	1	1	1	1	0	1	2
93	0	0	1	1	0	.	0	0	1	2
94	1	2	0	0	0	.	0	0	0	2
95	0	0	1	1	0	.	0	0	1	2
96	1	1	0	0	0	.	0	0	0	2
97	0	0	1	1	0	.	0	0	1	2
98	0	0	1	1	0	.	0	0	1	2
99	0	0	1	1	0	.	0	0	1	2
100	1	2	0	0	0	.	0	0	0	2

	ocv	doses	ors	meo	hosp	time	referal	dead	loperamide	cel
101	0	0	1	1	1	1	1	0	1	2
102	0	0	1	0	1	2	1	0	1	2
103	1	1	0	0	0	.	0	0	0	2
104	1	1	0	0	0	.	0	0	0	2
105	0	0	1	0	1	2	1	0	1	2
106	1	2	0	0	0	.	0	0	0	2
107	1	1	0	0	0	.	0	0	0	2
108	1	1	1	0	1	1	1	0	0	2
109	1	1	0	0	0	.	0	0	0	2
110	0	0	1	1	0	.	0	0	1	2
111	0	0	1	1	0	.	0	0	1	2
112	0	0	1	1	1	1	1	0	1	2
113	0	0	1	1	0	.	0	0	1	2
114	0	0	1	0	1	2	1	0	1	2
115	0	0	1	1	0	.	0	0	0	2
116	0	0	1	1	0	.	0	0	1	2
117	0	0	1	1	0	.	0	0	1	2
118	0	0	1	1	0	.	0	0	1	2
119	1	2	0	0	0	.	0	0	0	2
120	1	1	0	0	0	.	0	0	0	2
121	0	0	1	0	1	2	1	0	1	2
122	0	0	1	0	1	2	1	0	1	2
123	1	2	0	0	0	.	0	0	0	2
124	0	0	1	0	1	2	1	0	0	2
125	0	0	1	0	1	2	1	0	1	2
126	0	0	1	1	1	1	1	0	1	2
127	0	0	1	0	1	2	1	0	0	2
128	0	0	1	0	1	2	1	0	1	2
129	1	1	1	1	0	.	0	0	0	1
130	0	0	1	0	1	1	1	0	1	1
131	0	0	1	1	0	.	0	0	1	2
132	0	0	1	0	1	2	1	0	1	1
133	0	0	1	0	1	2	1	0	1	1
134	0	0	1	0	1	2	1	0	1	1
135	0	0	1	1	1	1	1	0	1	1
136	0	0	1	0	1	2	1	0	0	1
137	0	0	1	1	1	1	1	0	1	1
138	0	0	1	1	1	1	1	0	1	2
139	0	0	1	0	1	1	1	0	0	2
140	0	0	1	1	1	1	1	0	1	1
141	0	0	1	1	1	1	1	0	1	1
142	0	0	1	1	0	.	0	0	1	1
143	0	0	1	1	0	.	0	0	1	2
144	1	1	0	0	0	.	0	0	0	2
145	1	2	0	0	0	.	0	0	0	1
146	0	0	1	1	1	1	1	0	1	2
147	0	0	1	1	1	1	1	0	1	2
148	0	0	1	1	1	1	1	0	1	2
149	0	0	1	1	1	1	1	0	0	2
150	0	0	1	1	1	1	1	0	1	1

	ocv	doses	ors	meo	hosp	time	referal	dead	loperamide	cel
151	0	0	1	0	1	2	1	0	0	1
152	0	0	1	1	1	1	1	0	1	2
153	0	0	1	1	1	1	1	0	1	1
154	0	0	1	0	1	2	1	0	0	1
155	1	2	1	1	1	1	1	0	1	2
156	1	1	0	0	0	.	0	0	0	2
157	0	0	1	1	0	.	0	0	0	2
158	1	2	1	1	1	1	1	0	1	2
159	1	2	1	0	1	1	1	0	1	2
160	0	0	1	1	0	.	0	0	0	2
161	1	2	1	0	1	1	1	0	1	2
162	1	1	0	0	0	.	0	0	0	1
163	0	0	1	1	0	.	0	0	0	2
164	1	2	1	1	0	.	0	0	1	2
165	0	0	1	0	1	2	1	0	0	2
166	1	1	1	0	1	1	1	0	1	2
167	0	0	1	0	1	1	1	0	0	1
168	0	0	1	1	0	.	0	0	1	2
169	0	0	1	1	1	1	1	0	0	2
170	0	0	1	1	1	1	1	0	1	2
171	0	0	1	1	1	1	1	0	0	2
172	0	0	1	1	0	.	0	0	0	2
173	0	0	1	1	0	.	0	0	1	2
174	0	0	1	1	1	1	1	0	1	2
175	0	0	1	1	1	1	1	0	0	2
176	0	0	1	1	1	1	1	0	0	2
177	1	1	1	0	1	1	1	0	0	2
178	0	0	1	0	1	1	1	0	1	2
179	0	0	1	1	0	.	0	0	1	1
180	0	0	1	1	0	.	0	0	1	2
181	0	0	1	1	0	.	0	0	0	2
182	0	0	1	1	0	.	0	0	0	1
183	1	2	1	0	1	1	1	0	1	1
184	0	0	1	1	0	.	0	0	0	2
185	0	0	1	1	0	.	0	0	1	2
186	0	0	1	1	0	.	0	0	1	2
187	1	2	1	0	1	1	1	0	0	2
188	0	0	1	1	1	1	1	0	1	2
189	0	0	1	1	1	1	1	0	0	2
190	0	0	1	1	1	1	1	0	1	2
191	0	0	1	0	1	1	1	0	1	1
192	0	0	1	1	1	1	1	0	0	2
193	0	0	1	1	1	1	1	0	1	2
194	1	1	1	0	1	1	1	0	0	2
195	0	0	1	1	0	.	0	0	1	2
196	0	0	1	0	1	1	1	0	1	1
197	0	0	1	1	0	.	0	0	0	1
198	0	0	1	1	0	.	0	0	1	1
199	1	1	1	0	1	1	1	0	0	2
200	1	2	1	0	1	1	1	0	1	2

	ocv	doses	ors	meo	hosp	time	referal	dead	loperamide	cel
201	1	1	1	1	0	.	0	0	0	1
202	1	2	1	0	1	1	1	0	1	2
203	1	2	0	0	0	.	0	0	0	1
204	1	1	0	0	0	.	0	0	0	2
205	1	1	0	0	0	.	0	0	0	2
206	1	1	0	0	0	.	0	0	0	2
207	1	1	0	0	0	.	0	0	0	1
208	1	1	0	0	0	.	0	0	0	1
209	1	1	0	0	0	.	0	0	0	1
210	1	1	0	0	0	.	0	0	0	2
211	0	0	1	0	1	1	1	0	1	1
212	1	2	0	0	0	.	0	0	0	1
213	1	1	0	0	0	.	0	0	0	2
214	1	1	0	0	0	.	0	0	0	2
215	0	0	1	1	1	1	1	0	1	1
216	0	0	1	1	1	1	1	0	1	2
217	0	0	1	1	1	1	1	0	0	2
218	0	0	1	1	1	1	1	0	0	2
219	0	0	1	0	1	2	1	0	1	2
220	0	0	1	0	1	2	1	0	1	2
221	0	0	1	0	1	2	1	0	1	2
222	0	0	1	0	1	2	1	0	1	2
223	1	2	1	0	1	1	1	0	1	2
224	1	2	1	0	1	1	1	0	0	2
225	1	1	1	0	1	1	1	0	0	2
226	1	1	1	1	0	.	0	0	0	2
227	1	1	1	0	1	1	1	0	1	2
228	1	1	1	0	1	1	1	0	1	2
229	1	1	1	1	0	.	0	0	0	1
230	1	1	1	1	0	.	0	0	0	1
231	1	1	1	0	1	1	1	0	1	2
232	1	1	1	0	1	1	1	0	0	2
233	1	1	1	0	1	1	1	0	1	2
234	1	2	0	0	0	.	0	0	0	2
235	0	0	1	1	0	.	0	0	0	2
236	1	2	0	0	0	.	0	0	0	2
237	1	2	0	0	0	.	0	0	0	2
238	1	1	0	0	0	.	0	0	0	2
239	1	1	0	0	0	.	0	0	0	2
240	1	2	0	0	0	.	0	0	0	2
241	1	2	0	0	0	.	0	0	0	2
242	1	2	0	0	0	.	0	0	0	2
243	1	2	0	0	0	.	0	0	0	2
244	1	2	0	0	0	.	0	0	0	2
245	1	2	0	0	0	.	0	0	0	2
246	1	2	0	0	0	.	0	0	0	2
247	0	0	1	0	1	1	1	0	1	2
248	0	0	1	0	1	1	1	0	1	2
249	0	0	1	0	1	1	1	0	1	2
250	0	0	1	0	1	1	1	0	1	2

	ocv	doses	ors	meo	hosp	time	referal	dead	loperamide	cel
251	0	0	1	0	1	1	1	0	1	2
252	0	0	1	0	1	1	1	0	1	1
253	1	1	1	0	1	1	1	0	0	2
254	1	1	1	1	0	.	0	0	0	1
255	0	0	1	1	0	.	0	0	0	2
256	1	1	1	0	1	1	1	0	1	2
257	0	0	1	1	0	.	0	0	1	2
258	0	0	1	0	1	2	1	0	1	2
259	1	1	1	0	1	1	1	0	0	2
260	1	1	1	0	1	1	1	0	0	2
261	1	1	1	0	1	1	1	0	1	2
262	1	1	1	0	1	1	1	0	1	2
263	1	1	1	0	1	1	1	0	1	2
264	0	0	1	0	1	2	1	0	1	2
265	1	1	1	0	1	1	1	0	0	2
266	1	1	1	0	1	1	1	0	1	2
267	1	1	1	0	1	1	1	0	1	2
268	0	0	1	0	1	2	1	0	0	2
269	0	0	1	0	1	2	1	0	1	1
270	0	0	1	0	1	2	1	0	0	1
271	0	0	1	1	0	.	0	0	1	2
272	0	0	1	0	1	1	1	0	1	1
273	0	0	1	0	1	1	1	0	1	1
274	0	0	1	0	1	1	1	0	1	1
275	0	0	1	0	1	1	1	0	1	1
276	0	0	1	0	1	1	1	0	1	1
277	0	0	1	0	1	1	1	0	1	2
278	0	0	1	0	1	1	1	0	1	2
279	1	1	1	1	0	.	0	0	0	1
280	1	1	1	1	0	.	0	0	0	1
281	1	1	1	1	0	.	0	0	1	1
282	1	2	1	1	0	.	0	0	0	1
283	1	2	1	1	0	.	0	0	0	1
284	1	1	1	0	1	1	1	0	0	2
285	1	2	1	1	0	.	0	0	0	1
286	1	2	1	1	0	.	0	0	0	1
287	1	2	1	1	0	.	0	0	0	1
288	1	2	1	0	1	1	1	0	0	2
289	1	2	1	0	1	1	1	0	0	2
290	0	0	1	1	0	.	0	0	0	2
291	1	1	1	1	0	.	0	0	0	1
292	1	1	1	0	1	1	1	0	0	2
293	1	1	1	0	1	1	1	0	0	1
294	1	1	1	1	0	.	0	0	0	1
295	0	0	1	0	1	1	1	0	1	1
296	0	0	1	0	1	1	1	0	1	1
297	0	0	1	0	1	1	1	0	1	1
298	0	0	1	0	1	1	1	0	1	2
299	0	0	1	0	1	1	1	0	1	2
300	0	0	1	0	1	1	1	0	1	2

	ocv	doses	ors	meo	hosp	time	referal	dead	loperamide	cel
301	0	0	1	0	1	1	1	0	1	1
302	0	0	1	0	1	1	1	0	1	1
303	0	0	1	0	1	1	1	0	1	1
304	0	0	1	0	1	1	1	0	1	1
305	0	0	1	0	1	1	1	0	1	1
306	0	0	1	0	1	1	1	0	1	1
307	0	0	1	0	1	1	1	0	1	1
308	0	0	1	0	1	1	1	0	1	2
309	0	0	1	0	1	1	1	0	1	2
310	0	0	1	0	1	1	1	0	1	2
311	0	0	1	0	1	1	1	0	1	1
312	0	0	1	0	1	1	1	0	1	1
313	1	1	1	0	1	1	1	0	0	1
314	1	2	1	0	1	1	1	0	1	2
315	1	2	1	0	1	1	1	0	1	2
316	1	2	1	0	1	1	1	0	1	1
317	1	2	1	0	1	1	1	0	1	2
318	1	2	1	0	1	1	1	0	1	2
319	1	1	1	0	1	1	1	0	1	2
320	1	2	1	0	1	1	1	0	1	2
321	1	1	1	0	1	1	1	0	1	1
322	1	2	1	0	1	1	1	0	1	1
323	1	2	1	0	1	1	1	0	1	1
