# Supplementary material for: Effectiveness of a reactive oral cholera vaccination during a cholera outbreak at the Douala New-Bell Central Prison in Cameroon
Source: PLoS Negl Trop Dis. 2025 Dec 29;19(12):e0013870. doi: 10.1371/journal.pntd.0013870 (PMC12774372; doi:10.1371/journal.pntd.0013870)
Supplement: S2 File — (PDF) [file pntd.0013870.s002.pdf]

```
use "C:\Users\DS NKONGSAMBA\Documents\RESEARCH PROPOSALS\OCV in prison 1.dta"
describe
summ
destring, replace
gen ages=age
recode ages min/19.99=0 20/max=1
tab sex ocv, col chi
tab ages ocv, col chi
su age, d
swilk age
tab penal ocv, col chi
tab cel ocv, col chi
tab clinical ocv, col chi
tab vomit ocv, col chi
tab ocv
ci prop ocv
ci prop hosp
tab hosp
tab hosp ocv, col chi
tab hosp doses, col chi
nptrend hosp, group(doses) carmitage
tab hosp ages, col chi
tab hosp sex, col chi
tab hosp penal, col chi
tab hosp cel, col chi
tab hosp referal, col chi
tab hosp vomit, col chi
logistic hosp i.doses i.ages i.sex i.penal i.cel i.vomit
logistic hosp i.doses i.ages i.sex i.penal i.cel
logistic hosp i.doses i.sex i.penal i.cel
logistic hosp i.doses i.sex i.cel
```
