# Supplementary material for: Effectiveness of a reactive oral cholera vaccination during a cholera outbreak at the Douala New-Bell Central Prison in Cameroon
Source: PLoS Negl Trop Dis. 2025 Dec 29;19(12):e0013870. doi: 10.1371/journal.pntd.0013870 (PMC12774372; doi:10.1371/journal.pntd.0013870)
Supplement: Attachment — Submitted filename: My_Review.pdf [file pntd.0013870.s003.pdf]

Confidential Review for PNTD-D-25-01221

Reviewer for PLOS Neglected Tropical Diseases

Manuscript Number: PNTD-D-25-01221
Title: Effectiveness of a Reactive Oral Cholera Vaccination during a cholera outbreak at the Douala New-Bell Central Prison in Cameroon
Journal: PLOS Neglected Tropical Diseases
Overall Recommendation: Major Revision
I thank the authors for conducting this important and timely study on the effectiveness of oral cholera vaccination (OCV) in a prison setting. Research focusing on vulnerable and often neglected groups, such as incarcerated individuals, is essential for fair public health. The manuscript fills a clear gap in the literature. The finding that reactive OCV can significantly reduce cholera-related hospitalizations in this challenging environment is both new and highly relevant for outbreak response policy.
The study is generally well-structured. The use of logistic regression is suitable for the research question. The subgroup analyses showing greater protection for females and VIP inmates are particularly insightful. They highlight the important role of improved living conditions along with vaccination.
However, several major methodological concerns need clarification and significant revision to enhance the validity of the conclusions and the overall impact of the work.

Major Points

1. Critical Issue: Temporal Relationship and Reverse Causality

The retrospective design raises the possibility of bias from **reverse causality**. It's possible that inmates who were already in the incubation period or feeling unwell during the vaccination campaign were less likely to receive the vaccine. If these individuals later developed severe cholera that needed hospitalization, it would skew the results, making the vaccine seem more effective than it actually is.

Suggestions:

- The authors should discuss this limitation in much greater depth in the Discussion section.
- A **sensitivity analysis** is highly recommended. For instance, the authors could re-run their models after excluding cholera cases that occurred within the first 7 or 14 days post-vaccination. This would help assess the robustness of the vaccine effectiveness estimate against this specific bias.

2. Outcome Definition and Specificity

The main outcome is "cholera-related hospitalization." The manuscript mentions that laboratory confirmation was available "where available," but it does not specify the proportion of cases that were laboratory-confirmed.
Suggestions:
The authors need to clearly state what percentage of hospitalized cases were confirmed by culture, PCR, or rapid diagnostic test.
If a large number of cases were based on clinical diagnosis alone, the potential for misclassification, such as from other severe diarrheal illnesses like rotavirus or ETEC, should be noted as a limitation. This could affect the reported vaccine effectiveness.

3. Clarity of Sampling Methodology and Population

While a stratified sampling strategy is described, the process of selecting the final 323 participants from the total prison population of nearly 4,000 is not fully clear.

Suggestions:

- A more detailed description of the sampling process is needed. For example, were all eligible inmates within the strata listed and then a random sample selected?
- Including a **participant flow diagram** (for example, a CONSORT-type diagram for observational studies) would greatly improve clarity and show how representative the study sample is.

4. Presentation and Interpretation of Results in Table 2

The presentation of data in Table 2 is currently confusing and requires correction.

Suggestions:

- The column header ”Number of hospitalisations n (%)” is misleading. The numbers listed (e.g., 63, 27, 47, 20) seem to represent the total number of individuals in each exposure subgroup, not the number hospitalized. The percentages (e.g., 57.27%) likely show the proportion hospitalized within that subgroup.
- The table should be relabeled for clarity. A suggested header for the first data column is: ”Total N in subgroup (N hospitalized,
- The text (Results, Page 14) states that two doses are ”slightly more effective” than one. However, the adjusted Odds Ratios (aOR 0.39 vs. 0.37) are almost the same, with confidence intervals that heavily overlap. This claim is not supported by the data and should be reworded to show the uncertainty (e.g., ”While two doses showed a non-significant trend towards greater protection...”).

Minor Points

- **Introduction:** This section could be shortened to quickly focus on the issue of cholera in prison settings.
- **Abstract:** The sentence about female and VIP prisoners in the results section could be reworded for clarity. It should clearly indicate that this is a subgroup analysis of vaccine effectiveness.
- **Data Availability:** The statement ”All relevant data are within the manuscript and its Supporting Information files” is acknowledged. The uploaded ‘.dta‘ file supports this, which is good practice.

Conclusion

This manuscript presents an important public health intervention study. The main finding that OCV can lower severe cholera rates in a prison is significant. I recommend **Major Revision** to clarify potential reverse causality, case definitions, and sampling descriptions. Fixing these issues will greatly improve the manuscript and reinforce its contribution to the field. I look forward to reviewing a revised version.
