# Supplementary material for: Effectiveness of a reactive oral cholera vaccination during a cholera outbreak at the Douala New-Bell Central Prison in Cameroon
Source: PLoS Negl Trop Dis. 2025 Dec 29;19(12):e0013870. doi: 10.1371/journal.pntd.0013870 (PMC12774372; doi:10.1371/journal.pntd.0013870)
Supplement: Attachment — Submitted filename: Reverwer comment_cln.docx [file pntd.0013870.s004.docx]

**Manuscript title:** Effectiveness of a Reactive Oral Cholera Vaccination during a cholera outbreak at the Douala New-Bell Central Prison in Cameroon

**Review Comments**

**1. Abstract (pp. 2–3, lines 25–51)**

- Clarity of design: The phrase *“retrospective secondary data analysis”* could mislead reviewers, since effectiveness implies temporality. Clarify whether this is a retrospective cohort or cross-sectional analytic design.
- Wording: Correct “adjusted odd ratios” → “adjusted odds ratios.”
- Data transparency: Add denominators for vaccinated/unvaccinated participants (e.g., “20/110 vs 63/213 hospitalised”).
- Interpretation: Avoid causal language (“significantly reduce”) until temporality bias is addressed; use “associated with lower odds.”
- Line 45–46: The conclusion *“may not be enough to control cholera”* should be tempered—tie to concurrent WASH improvements.

**2. Introduction (pp. 3–4, approx. lines 52–105)**

- The background establishes relevance but omits literature on *prison settings*. add one or two prior OCV studies in confined institutions.
- Add a quantitative statement of global cholera burden to frame importance (e.g., WHO 2024 est.).
- Clarify research gap: *“No prior published data on OCV effectiveness among incarcerated populations in Africa.”*

**3. Methods (pp. 4–7, lines 126–226)**

a. Study design and population

- Line 144–151: The term *“institutional cross-sectional study”* contradicts causal inference, relabel as *retrospective cohort* or *analytic cross-sectional* depending on temporal data.
- Provide inclusion/exclusion criteria (age ≥ ? ; duration of incarceration > ? ).

b. Sampling

- Lines 156–165: Stratified sampling by gender/housing type, state exact strata sizes and sampling fractions.

c. Exposure definition

- Lines 93–100 & 172–179: Specify vaccine product (Euvichol® vs Euvichol-Plus®), dose interval, and campaign dates; define *“vaccinated”* as ≥ 7 days post-dose.

d. Outcome definition

- Lines 179–193: Define “cholera-related hospitalisation”: was it based on IV rehydration, ≥ 24 h stay, or clinician judgment?

f. Sample-size calculation

- Lines 166–171: Discuss how realised allocation (~2:1 unvaccinated:vaccinated) affects power.

**4. Results (pp. 7–10, lines 228–275)**

a. Descriptive statistics

- Lines 228–237: Only 34.9 % vaccinated, though campaign coverage was > 80 %, explain discrepancy (missing records or population turnover).
- Provide a flow diagram: eligible → sampled → analysed.

b. Table 2 (lines 255–266)

- Denominators and percentages inconsistent (e.g., 20/110 ≠ 41.7 %). Recalculate and show *n/N (%)* for each dose level.
- Separate baseline characteristics (Table 1) from outcome (Table 2).
- Add absolute risk difference and risk ratio alongside ORs.
- Add p-trend test for 0, 1, 2 doses.

c. Dose–response claim

- Lines 264–268: The aORs (0.39 vs 0.37) overlap; do not assert a gradient without statistical test.

e. Figures

- Add epidemic curve (onset dates) with vaccination timeline to confirm exposure precedes outcomes.

**5. Discussion (pp. 10–12, lines 276–303)**

- Lines 276–287: Re-structure to mirror objectives: (1) principal finding, (2) interpretation vs prior studies, (3) programmatic implications.
- Discuss potential immortal-time bias and reverse causation explicitly; describe sensitivity analysis excluding cases with onset ≤ 7 days post-vaccination.
- Integrate WASH context—improvements in VIP/female blocks could confound VE estimates.
- Refrain from over-generalising to “all prisons” without noting structural differences.
- Acknowledge limited lab confirmation, small sample size, and reliance on secondary records.
- Expand implications: integrate OCV with WASH and surveillance upgrades in carceral settings.

6. Tables, Figures, and Supplementary Files (pp. 13–16)

- Tables: Ensure denominators consistent; add clear titles (“Baseline characteristics by vaccination status,” “Risk of hospitalisation by OCV dose”).
- Figures: Epidemic curve and dose-response plot recommended.
- Supporting data (p. 16 lines 40–46): Include variable dictionary and analysis code for transparency.
