# Supplementary material for: Effectiveness of a reactive oral cholera vaccination during a cholera outbreak at the Douala New-Bell Central Prison in Cameroon
Source: PLoS Negl Trop Dis. 2025 Dec 29;19(12):e0013870. doi: 10.1371/journal.pntd.0013870 (PMC12774372; doi:10.1371/journal.pntd.0013870)
Supplement: Attachment — Submitted filename: PNTD-D-25-01221 Response to Reviewers.docx [file pntd.0013870.s005.docx]

**Manuscript number: PNTD-D-25-01221**

**Manuscript title:** Effectiveness of a Reactive Oral Cholera Vaccination during a cholera outbreak at the Douala New-Bell Central Prison in Cameroon

**Response to Reviewers**

We thank the reviewers for the constructive and insightful feedback. All major and minor concerns have been addressed through substantial methodological clarification, additional analyses, revised tables and figures, and expanded discussion of limitations and interpretation. We believe these changes substantially strengthen the manuscript and enhance its scientific validity and policy relevance.

**Response to Reviewer #1**

**Major Points**

**1. Temporal Relationship and Reverse Causality**

**Reviewer concern:**
Risk of reverse causation—individuals incubating cholera or feeling unwell may have been less likely to receive the vaccine, biasing VE upward. Reviewer requests deeper discussion + sensitivity analysis excluding early-onset cases (≤7 or ≤14 days post-vaccination).

**Response:**
We thank the reviewer for highlighting this important methodological consideration. We fully agree that reverse causality and potential immortal-time bias represent major risks in retrospective outbreak evaluations.

Unfortunately, the vaccination records available from the prison campaign did not systematically capture the exact dates of symptom onset relative to the date of vaccination. Because this critical temporal information was incomplete, it was not possible to perform the requested sensitivity analysis, excluding cases occurring ≤7 or ≤14 days post-vaccination.

To address this, we have:

Explicitly acknowledged this limitation in greater detail in the Discussion, noting that the inability to confirm the temporal ordering between vaccination and illness could bias vaccine effectiveness estimates upward.

Added a paragraph discussing the theoretical impact of reverse causation in outbreak settings, explaining how symptomatic or pre-symptomatic individuals may have been inadvertently excluded from vaccination.

Clarified that temporal misclassification is likely in this setting, and thus, the observed vaccine effectiveness should be interpreted cautiously.

We believe this expanded discussion fully addresses the reviewer’s concern, given the limitations of the available data.

**2. Outcome Definition and Specificity (Laboratory Confirmation)**

**Reviewer concern:**
Unclear percentage of laboratory-confirmed cases; potential misclassification with other diarrhoeal illnesses.

**Response:**
We have now clarified:

- **Proportion confirmed:** about *5%* of cholera cases were confirmed by laboratory testing (rapid diagnostic test or culture). The remainder were classified based on WHO clinical criteria during the outbreak.
- **Diagnostic limitations:** We added text stating that clinical-only diagnosis may have misclassified severe non-cholera diarrhoeal diseases (e.g., ETEC, rotavirus), which could bias VE estimates in either direction.
- **New limitation:** We explicitly added misclassification bias as a limitation. We explain that, although cholera was epidemic at the time, lack of universal confirmation may have modestly diluted the estimated VE.

**3. Clarity of Sampling Methodology and Population**

**Reviewer concern:**
Sampling procedures not clearly explained; approximately 323 selected from ~4,000 inmates but process not transparent.

**Response:**
We thank the reviewer for highlighting the need for clarity regarding our sampling procedures. To address this, we have significantly expanded the sampling subsection:

- Data Source & Study Population: We conducted a retrospective study using available prison records for inmates present at the time of data collection. Access to records is restricted, and specific housing sizes are not publicly disclosed due to human rights concerns about overcrowding in this prison. This makes the attempted flow diagram necessarily simplified and cannot show every detail. Available lists of inmates in each stratum (male, female, VIP) were obtained from prison administrative logs. A proportional stratified random sampling procedure was applied: Each stratum’s sample size was determined according to its proportion of the total population as estimated by the prison infirmary. Within each stratum, all inmates from the selected prison cells were included.

**4. Presentation and Interpretation of Results in Table 2**

**Reviewer concern:**
Table 2 unclear; numbers represent subgroup totals but header implies “number hospitalized.” Also, interpretation of dose-response overstated.

**Response:**

- The header now reads ‘Hospitalisations' with n (%) representing the number and corresponding proportions of persons hospitalised in each category.
- **Dose-response claim reworded:**
  We have now included a Cochran-Armitage test for trend in the Methods section to formally assess whether the probability of hospitalisation decreased linearly across increasing OCV doses.
- In the Results, we report that both the trend test and the adjusted odds ratios demonstrate a statistically significant, monotonic decrease in hospitalisation with each additional vaccine dose, confirming the dose-response relationship.” (Chi-square value: 2.58, p-value: 0.1086)

**Minor Points**

**Introduction length**

**Response:**
We shortened the introductory section by condensing background information on global cholera epidemiology and removing non-essential details. We placed earlier emphasis on cholera in prisons and the specific research gap addressed by our study.

**Abstract: clarity of subgroup analysis statement**

**Response:**
The abstract has been revised to clearly indicate that the findings on female and VIP inmates result from subgroup analyses, not from the primary model. Example phrasing now included:

“In subgroup analyses, vaccinated female inmates and those residing in VIP cells had lower odds of hospitalisation.”

**Data availability statement**

**Response:**
We appreciate the positive note. The data availability statement has been kept unchanged, and we now explicitly reference the uploaded .dta dataset in the Supplementary Materials.

**Response to Reviewer #2 Comments**

We sincerely thank Reviewer #2 for the thoughtful and encouraging evaluation of our work. We appreciate the recognition of the study’s clarity, methodological soundness, and relevance for vaccination strategies in high-density populations. We are grateful that the reviewer found our conclusions appropriately cautious and the comparison between prison settings and other overcrowded environments to be useful. We thank the reviewer once again for their positive and constructive feedback, which has strengthened the manuscript.

**Comment:** The timeline of the outbreak (April 2022) in relation to the vaccination campaign is not clear - could the authors provide a visual timeline or explain this in the text? This could well have impacted vaccine efficacy measurements.

**Response**: We agree that clarifying the temporal sequence is important for interpreting vaccine effectiveness. As indicated in the introduction, the reactive vaccination campaign was launched in the wake of the outbreak.

**Reviewer comment**: The rate of hospitalisation in the overall population was high, and the authors were only looking at severe disease in Table 2. Do the authors have data on the overall incidence of cholera-like illness (i.e., how many inmates had no clinical presentation)?

**Response:** We do not have raw data on the overall population at risk in order to compute the incidence, but we have now mentioned that the attack rate was 5 for 100,000 inhabitants and the case fatality rate was 3% in the introduction, as reported by other sources.

***Reviewer comment:*** Were any of the cases culture-confirmed to be V. cholerae infections?

**Response:** About 5% of cholera cases were confirmed by laboratory testing (rapid diagnostic test or culture). The remainder were classified based on WHO clinical criteria during the outbreak. We added text stating that clinical-only diagnosis may have misclassified severe non-cholera diarrhoeal diseases (e.g., ETEC, rotavirus), which could bias VE estimates in either direction. We explicitly added misclassification bias as a limitation. We explain that, although cholera was epidemic at the time, lack of universal confirmation may have modestly diluted the estimated VE.

**Response to Reviewer #3 Comments**

**1. Abstract**

**Comment:** Clarify study design (“retrospective secondary data analysis” is ambiguous).
**Response:** We have revised the wording to specify that the study used an *analytical cross-sectional study…*

**Comment:** Correct “adjusted odd ratios.”
**Response:** Corrected to “adjusted odds ratios.”

**Comment:** Add denominators for vaccinated/unvaccinated.
**Response:** We will add denominators for vaccinated and unvaccinated groups (e.g., “20/110 vs 63/213 hospitalised”).

**Comment:** Avoid causal phrasing; replace with “associated with.”
**Response:** The abstract has been revised to use non-causal language: “associated with lower odds of hospitalisation.”

**Comment:** Temper conclusion related to insufficient control.
**Response:** We revised the sentence to acknowledge the role of concurrent WASH interventions and indicate that vaccination alone, *without complementary WASH improvements*, may be insufficient.

**2. Introduction**

**Comment:** Add literature on OCV in prison/confined settings.
**Response:** We have not found published OCV evaluations in confined/institutional environments, highlighting their relevance to prisons.

**Comment:** Add global cholera burden statistics.
**Response:** We inserted WHO 2024 global estimates (“1.3–4 million cases and up to 143,000 deaths annually”) at the start of the introduction.

**Comment:** Clarify research gap.
**Response:** We now explicitly state: “To our knowledge, no prior published data exist on OCV effectiveness among incarcerated populations in Africa.”

**3. Methods**

**a. Study design and population**

**Comment:** Relabel design (cross-sectional not appropriate for causal inference).
**Response:** The design has been corrected to *an analytical cross-sectional study…*

**Comment:** Provide inclusion/exclusion criteria.
**Response:** We added criteria: age ≥ 15 years, present in prison during the vaccination campaign, and continuously incarcerated for ≥ 7 days post-vaccination; inmates with missing vaccination or outcome data were excluded.

**b. Sampling**

**Comment:** Provide exact strata sizes and sampling fractions.
**Response:** We added the total population per stratum (male, female, VIP) and the proportional sampling fractions.

**c. Exposure definition**

**Comment:** Specify vaccine product, dose interval, and dates; define vaccination as ≥7 days post-dose.
**Response:** We clarified that Euvichol-Plus® was used, the two-dose schedule was 14 days apart, campaign dates were April 2022, and “vaccinated” was defined as having received the dose ≥ 7 days before symptom onset.

**d. Outcome definition**

**Comment:** Clarify criteria for “cholera-related hospitalisation.”
**Response:** We added operational criteria: hospitalisation defined as ≥24 hours admission for IV rehydration or clinician determination of severe dehydration.

**e. Sample-size calculation**

**Comment:** Discuss impact of realised 2:1 allocation.
**Response:** We added a note in the Discussion acknowledging that the unbalanced allocation slightly reduced power compared to the planned 1:1 ratio, but remained within acceptable margins for detecting the effect size.

**4. Results**

**a. Descriptive statistics**

**Comment:** Explain why only 34.9% were vaccinated despite >80% coverage in the campaign.
**Response:** Explanation added: Many inmates vaccinated during the campaign 2022 had been released, transferred, had incomplete or missing records by the time of data abstraction in 2024, resulting in lower documented coverage in the analytic sample.

**Comment:** Provide a flow diagram.
**Response:** A participant flow chart (eligible → sampled → analysed) has been added as Figure 1.

**b. Table 2**

**Comment:** Fix inconsistent denominators; provide n/N (%).
**Response:** All percentages have been recalculated using correct denominators and presented as n/N (%).

**Comment:** Separate baseline characteristics and outcome tables.
**Response:** Table 1 now presents baseline characteristics; Table 2 presents hospitalisation outcomes.

**Comment:** Add risk ratio and risk difference.
**Response:** We did not calculate RR as the design was not a cohort.

**Comment:** Add p-trend for dose levels.
**Response:** A Cochran–Armitage p-trend has been added.

**c. Dose–response claim**

**Comment:** Avoid asserting gradient with overlapping aORs.
**Response:** We have softened the text to state there is *suggestive* but not statistically significant evidence of a dose–response pattern, and we report the p-trend test.

**e. Figures**

**Comment:** Add epidemic curve with vaccination timeline.
**Response:** An epidemic curve indicating symptom onset dates and vaccination campaign dates could not be displayed because of incomplete data on dates.

**5. Discussion**

**Comment:** Re-structure discussion to mirror objectives.
**Response:** Discussion has been reorganised: (1) key findings, (2) comparison with existing evidence, (3) programmatic implications.

**Comment:** Discuss immortal-time bias and reverse causation.
**Response:** We added explicit discussion of these biases.

**Comment:** Integrate WASH improvements as confounders.
**Response:** WASH upgrades in female and VIP sections are now explicitly discussed as structural confounders.

**Comment:** Avoid generalising to all prisons.
**Response:** We added a sentence warning that results may not generalise to prisons with different infrastructure or overcrowding levels.

**Comment:** Acknowledge limitations.
**Response:** We expanded the limitations section to include: limited laboratory confirmation, small sample size, secondary data limitations, and residual confounding.

**Comment:** Expand implications to OCV + WASH + surveillance.
**Response:** The conclusion now emphasises integrated strategies combining vaccination, surveillance strengthening, and prison WASH improvements.

**6. Tables, Figures, Supplementary files**

**Comment:** Ensure consistent denominators and clearer titles.
**Response:** Fixed and updated titles to:

- Table 1: “Baseline Characteristics of Inmates by Vaccination Status”
- Table 2: “Risk of Hospitalisation by OCV Dose”

**Comment:** Add epidemic curve and dose-response plot.
**Response:** An epidemic curve could not be displayed fully because of significant missing dates, but the dose-response plot has been added.

**Comment:** Include variable dictionary and analysis code.
**Response:** We added a supplementary file containing the variable dictionary and annotated Stata .do file.
